# Diagnostic Value of Rapid On-Site Evaluation for CT-Guided Percutaneous Fine Needle Aspiration in the Diagnosis of Pulmonary Occupying Lesions

**DOI:** 10.1155/2020/9842768

**Published:** 2020-11-17

**Authors:** Tian-Feng Peng, Tao Ren, Han-Sheng Wang, Zhe-Xiang Feng, Mei-Fang Wang

**Affiliations:** ^1^The First Clinical Medical College, Hubei University of Medicine, Shiyan, Hubei 442000, China; ^2^Department of Respiratory and Critical Care Medicine, Taihe Hospital, Hubei University of Medicine, Shiyan, Hubei 442000, China

## Abstract

**Objective:**

Rapid on-site evaluation (ROSE) is an effective and efficient auxiliary examination, but its value for CT-guided percutaneous fine-needle aspiration (FNA) in the diagnosis of pulmonary occupying lesions is unclear. This study is aimed at evaluating the clinical utility of ROSE for CT-guided percutaneous FNA.

**Methods:**

We reviewed 234 patients from September 2018 to April 2019. The result using ROSE was compared with the final pathological diagnosis of CT-guided percutaneous FNA, and we also compared the complications between the ROSE group and the NO-ROSE group. The final pathological diagnosis results served as the gold standard. We also analyzed the diagnostic rate of FNA and the sensitivity, specificity, positive predictive value (PPV), and negative predictive value (NPV) of malignancy. The correlation between diverse pathological types of lung cancer was also taken into consideration.

**Results:**

In total, 132 patients underwent CT-guided percutaneous FNA with ROSE (ROSE group), and 102 did not (NO-ROSE group). The diagnostic rate, sensitivity, specificity, PPV, and NPV of the ROSE group were 91.6%, 89.1%, 94.1%, 93.4%, and 90.1%, respectively. The complication rates of the ROSE group and the NO-ROSE group were 8.33% and 16.67%, respectively. This difference was not statistically significant (*P* > 0.05). In subsets of adenocarcinoma (AC) and small cell lung cancer (SCLC) patients, the ROSE result was highly consistent with the final pathological result.

**Conclusion:**

CT-guided percutaneous FNA combined with ROSE has a high diagnostic rate, sensitivity, and specificity for pulmonary occupying lesions and an acceptable rate of complications. This method is worthy of wide use given its high efficiency and safety.

## 1. Introduction

Lung cancer, as a common malignancy worldwide, seriously threatens human life and health. Lung cancer accounts for 11.6% of all new cancer cases and 18.4% of cancer-related death [1]. Lung cancer is the most common cause when chest CT images indicated lung-occupying lesions, but other diseases, including metastasis, tuberculosis, and pulmonary infection, should also be taken into account. Different conditions have different prognoses. For example, various lung cancers, which have similar etiologies, also exhibit significantly different prognoses based on pathologic type, genetic mutation, and stage. In view of the etiology of pulmonary occupying lesions, various methods, such as electronic bronchoscopy, percutaneous FNA, or surgery, are used to obtain pathological, cytological, and bacteriological evidence based on the patient's physical condition and lesion location [2]. CT-guided percutaneous FNA is a common method that is often used clinically given its low invasion and acceptable rate of complications. A pathologist examined tissues after they were obtained. The final pathological results served as the gold standard for etiological diagnosis. Rapid on-site evaluation (ROSE), an auxiliary technique applied in interventional pulmonology, is a real-time cytologic interpretation technology and sampling process [3–6]. Our study is aimed at investigating the value of ROSE in the clinical diagnosis of pulmonary occupying lesions during CT-guided percutaneous FNA.

## 2. Materials and Methods

### 2.1. Patients

The Institutional Review Board of the Taihe Hospital of Hubei University of Medicine approved this study. A total of 234 patients from Taihe Hospital of Hubei University of Medicine were analyzed retrospectively from September 2018 to April 2019 and divided into two groups. In total, 132 patients who underwent CT-guided percutaneous FNA formed the ROSE group, and 102 who did not comprise the NO-ROSE group. The following inclusion criteria were employed: (1) thoracic CT scan revealed space-occupying lesions in the lung; (2) preoperative examinations, such as electrocardiogram (ECG), routine blood and urine test, coagulation function, and thoracic enhanced CT scan were completed to determine whether patients tolerate CT-guided percutaneous FNA and patients without obvious contraindications; (3) patients and their families have been apprized of the risk of this operation and signed the informed consent before the operation. The following exclusion criteria were employed in the study: (1) patients with severe cardiorespiratory function failure, coagulation dysfunction, and other surgical contraindications or patients who were too weak to undergo this operation; (2) people who were allergic to anesthetics; (3) people who received a definite diagnosis by bronchoscopy or another examination.

### 2.2. CT-Guided Percutaneous FNA Procedure

**Figure 1:**
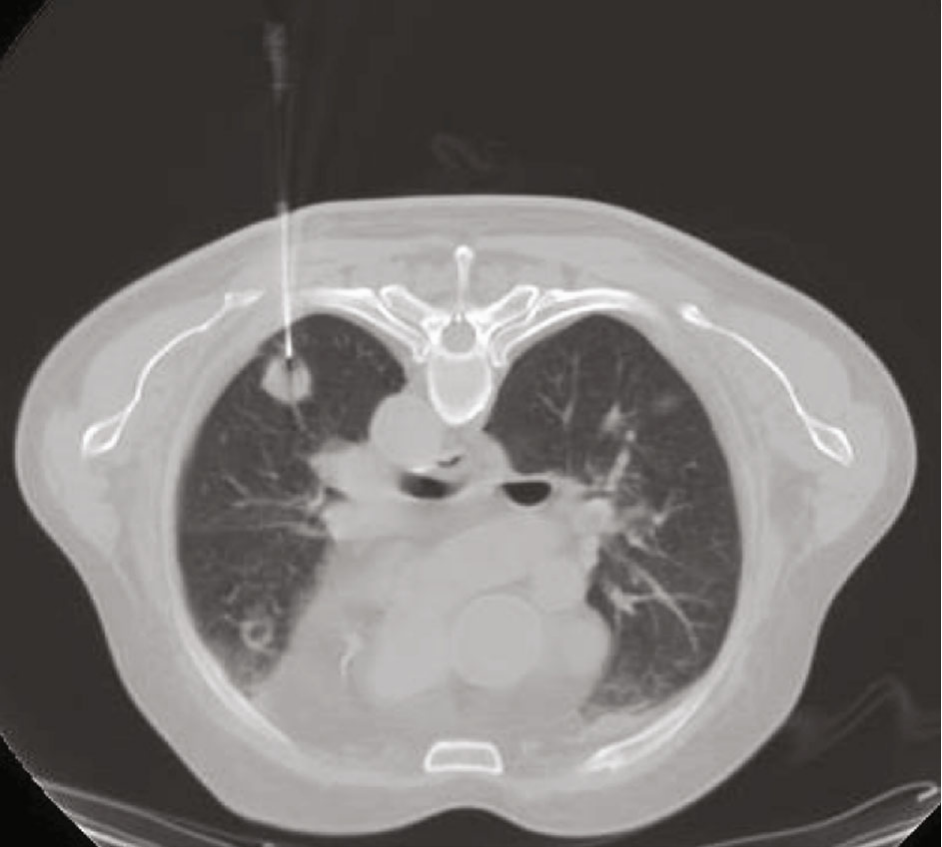
CT scan of lesions.

We ensured that preoperative examinations were completed to evaluate the surgical risk, and we clearly explained the surgical procedure and the risks to the patients and their families. Informed consent was obtained. Drinking and fasting were forbidden up to 4 hours before the operation to prevent intraoperative vomiting or aspiration. We established vein channels and performed ECG monitoring and finger pulse oxygen monitoring throughout the entire procedure. CT scan was performed to determine the location, size, and surroundings of lesions before the operation to select the best puncture point (Figure 1). Every patient was placed in an appropriate position to perform the operation. Position selection was dependent on the puncture site. A supine position was chosen for the upper lobe and hilum, a lateral position was selected for the middle lobe, and a prone position was selected for the basal and dorsal segments of the lower lobe. This position was chosen because it was convenient for physicians to perform the operation and was simultaneously tolerable for the patient. In addition, the position minimizes trauma to protect the lung. First, the operator provided a sterile sheet, disinfected the overlying skin, and injected lidocaine for local anesthesia. The puncture needle was placed at the puncture point for injection. Chest CT scanning was performed repeatedly to adjust the needle depth and position until the best site was selected. Then, the needle was removed, the puncture site was routinely disinfected, and proper pressure was applied to avoid bleeding. The patient was asked to return to the ward and remain in bed.

### 2.3. Rapid On-Site Evaluation Procedure

The aspirated materials in the ROSE group were smeared on-site using a rolling (printing) method after each puncture was performed by a physician from the Respiratory Function Room who received standardized training for 3 months. Other materials were placed into formalin for fixation and then submitted to the pathologist for follow-up work, including staining, smearing, and interpreting. The completed smear was stained with Diff-Quik dye as recommended by the WHO for 30-70 seconds and then interpreted immediately by a physician who had received standardized training of assessment of cytological characteristics under a microscope for 3 months.

### 2.4. Diagnostic Criteria

The diagnostic results of the ROSE group were compared with the final pathological results. If the results of the ROSE group were consistent with the final pathological results, the diagnostic results of the ROSE group served as the ultimate result. Alternatively, the final pathological results served as the final result.

### 2.5. Statistical Analysis

SPSS software version 22.0 (IBM Corp., Shiyan, Hubei, China) was used for statistical analysis. Correlations between the ROSE group and the final pathological results were assessed using the kappa statistic. A kappa value of ≤0.40 indicated poor agreement, 0.40–0.75 indicated moderate agreement, and ≥0.75 indicated excellent agreement. The sensitivity, specificity, positive predictive value (PPV), negative predictive value (NPV), and diagnostic accuracy of ROSE during CT-guided percutaneous FNA were derived according to the relevant final pathologic results. A *P* value < 0.05 was considered statistically significant.

## 3. Results

### 3.1. Clinical Data

We enrolled 234 patients in our study, including 132 patients who underwent CT-guided percutaneous FNA and 102 who did not. No statistically significant differences in demographic data were noted between the ROSE group and the NO-ROSE group (*P* > 0.05) (Table 1).

### 3.2. Cytological Characteristics of Smears by ROSE

**Figure 2:**
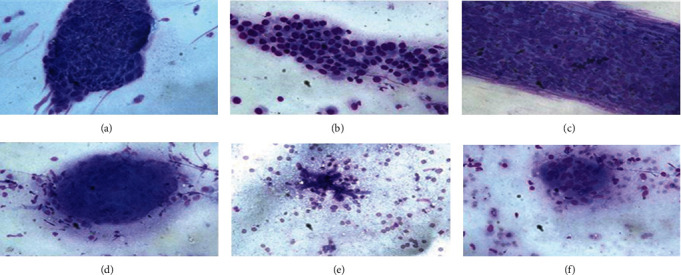
Cytological characteristics of ROSE for SCC, AC, SCLC, tuberculosis, Aspergillus, and Cryptococcus neoformans.

Smears of squamous cell carcinoma (SCC) were composed of mainly dispersed, often elongated, or spindle-shaped cells with dense cytoplasm and keratinization. Nuclei are often pyknotic or hyperchromatic with angulated contours. Small dyscohesive sheets of malignant cells with enlarged nuclei with nucleoli and coarse chromatin and fragments of dispersed keratinizing cells with dense cytoplasm and pyknotic nuclei were noted. Large tissue fragments composed of cells with enlarged nuclei with macronucleoli were also noted (Figure 2(a)). Adenocarcinoma manifests as obvious glandular differentiation or dyscohesive aggregates of cells with large nuclei, prominent nucleoli, and tumor cells with single intracytoplasmic vacuoles or globular secretory material indicate glandular differentiation (Figure 2(b)). Small cell lung carcinoma presents as small cells with a high N/C ratio, scant and poorly preserved cytoplasmic apoptosis, “salt and pepper” chromatin texture, and nuclear molding (Figure 2(c)). Tuberculosis appears as granular and amorphous debris with poorly preserved granulomas (Figure 2(d)). The organisms of aspergilloma exhibit uniform thickness and branching at acute angles. Septate hyphae show dichotomous branching at 45° and a diameter of 3–6 *μ*m (Figure 2(e)). Cryptococcus neoformans smears appear as a circular refracted substance in the granuloma with a thick capsule (Figure 2(f)).

### 3.3. Pathological Results of the ROSE and NO-ROSE Groups

The ROSE group comprised 132 patients, including 61 patients diagnosed with neoplasm (46.2%) and 71 patients diagnosed with noncancer-related disease (53.3%). The final pathological diagnosis of the ROSE group included 63 cases of neoplasm (47.7%) and 69 cases of noncancer-related disease (52.8%). Among the 102 patients in the NO-ROSE group, 54 cases of neoplasm (52.9%) and 48 cases of noncancer-related disease (47.1%) were diagnosed. The specific pathological types are shown in Table 2.

### 3.4. Comparison of CT-Guided Percutaneous FNA Combined with ROSE and Final Pathological Diagnosis for Different Types of Malignant and Pulmonary Infectious Diseases

The diagnostic rate, sensitivity, specificity, PPV, and NPV of malignancy in the ROSE group were 91.6%, 89.1%, 94.1%, 93.4%, and 90.1%, respectively (Table 3).

A statistically significant difference was noted between the ROSE result and the final pathological diagnosis for different types of malignancies (*χ*^2^ = 21.368, *P* < 0.05). An analysis of the diagnoses is presented in Table 4. Among them, 33 cases of adenocarcinoma were confirmed by both ROSE and pathology; the ROSE and pathological diagnosis results were very consistent (Kappa = 0.767, *P* < 0.001). The diagnostic rate of ROSE was lower than the pathological results (34.1% vs. 25.8%), and the difference was statistically significant (*P* = 0.003 < 0.05). The sensitivity, specificity, PPV, and NPV were 73.3% (33/45), 98.9% (86/87), 97.1% (33/34), and 87.7% (86/98), respectively. For squamous cell carcinoma, the ROSE and pathological results were moderately correlated (Kappa = 0.438, *P* < 0.001), with a sensitivity of 36.4% (4/11), specificity of 98.3% (119/121), PPV of 66.7% (4/6), and NPV of 94.4% (119/126). The difference in the diagnosis rate between ROSE and the pathological results was not statistically significant (*P* = 0.18 > 0.05). For small cell lung cancer, the ROSE and pathological diagnosis were in good agreement (Kappa = 1.000, *P* < 0.001), and the difference in the diagnosis rate between the ROSE results and the pathological results was not statistically significant (*P* > 0.05) (Table 5). In one case, the ROSE result and the final pathological result both provided a diagnosis of nonsmall cell lung cancer-not otherwise specified (NSCLC-NOS).

The comparison between the results of ROSE and the final pathological diagnosis of different types of lung infections is shown in Table 6. No statistically significant differences were noted between the ROSE results and the pathological results in the diagnosis of pulmonary infectious diseases (*χ*^2^ = 2.203, *P* = 0.374 > 0.05). In the ROSE group, the diagnostic rate for lung inflammation was 90.2% (119/132), the sensitivity was 80.0% (24/30), the specificity was 93.1% (95/102), the PPV was 77.4% (24/31), and the NPV was 94.1% (95/101). For tuberculosis, the diagnosis rate, sensitivity, specificity, PPV, and NPV were 90.9% (120/132), 95.8% (23/24), 89.8% (97/108), 67.6% (23/34), and 99.0% (97/98), respectively. For fungal infections, the diagnosis rate, sensitivity, specificity, PPV, and NPV were 98.5% (130/132), 33.3% (1/3), 100% (129/129), 100% (1/1), and 98.5% (129/131), respectively (Table 7).

### 3.5. Comparison of Complications between the ROSE Group and the NO-ROSE Group

**Figure 3:**
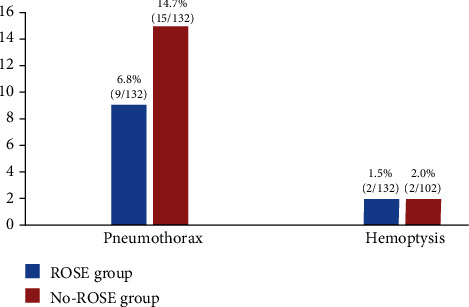
Complication rate in the ROSE group and the NO-ROSE group (*P* > 0.05).

The incidence of complications in the ROSE group was 8.3% (11/132), including pneumothorax in 6.8% of cases (9/132) and hemoptysis in 1.5% of cases (2/132). The incidence of complications in the NO-ROSE group was 16.7% (17/102), including pneumothorax in 14.7% cases (15/102), and hemoptysis in 2.0% of cases (2/102). No statistically significant differences were noted between the comparisons of two groups (*P* > 0.05) (Figure 3).

## 4. Discussion

It is imperative to confirm the diagnosis and implement relevant treatment as soon as possible in patients with pulmonary occupying lesions indicated by radiological imaging. CT-guided percutaneous FNA is used widely given its high diagnosis rate and acceptable complication rate in the clinic.

Compared with traditional flexible bronchoscopy, CT-guided percutaneous FNA is more beneficial in the diagnosis of peripheral lung lesions and offers a more accurate diagnosis of malignancy [7–11]. However, the success of the FNA significantly relies on the operator's proficiency and the location and size of the lesions. Lesion size is a critical factor for diagnostic accuracy, and the accuracy of the diagnosis is directly proportional to the reduction in the lesion diameter [12]. ROSE is an auxiliary examination used to assess the quality of samples. However, there are few domestic studies on the use of ROSE in CT-guided percutaneous FNA, and foreign studies have diverse conclusions. Some studies have evaluated punctured tissues after operation, so these studies are unable to assess the qualification and sufficiency of FNA tissue in real-time. However, the use of ROSE in this process can provide real-time guidance, improve diagnostic accuracy, reduce the risk of operation, and avoid unnecessary harm. ROSE should be applied to CT-guided FNA [11, 13–19]. Some studies indicate that the success of ROSE requires an experienced and united team; otherwise, more time will be wasted [15, 20, 21]. In addition, other studies have shown that on-site adequacy assessments may prolong the procedure, increase patient discomfort and anxiety, and waste precious resources. This study also showed that the 4-6 times was optimal for needling, and increasing the number did not significantly increase the diagnostic rate [22]. O'Malley et al. [23] assessed whether the obtained materials are adequate and suitable and are not affected by ROSE. No remarkable differences were noted between the ROSE group and the NO-ROSE group (*P* < 0.05), and rapid on-site assessment effectively saves time. Another prospective randomized controlled trial performed by Yarmus et al. [24] demonstrated that using ROSE does not improve the diagnostic rate and suggested that routine use of ROSE may not be necessary. Therefore, it remains unknown whether ROSE should be routinely applied in CT-guided FNA.

In our study, the ROSE group exhibited an increased diagnosis rate, sensitivity, and specificity for lung cancer, and the ROSE and pathological diagnosis results were consistent (Kappa = 0.833). This finding is consistent with the results of Wangsiricharoen et al. [25]. Further assessment of different pathological types of malignancy in our study showed that the results for AC and SCLC in the ROSE group were significantly correlated with the pathological diagnosis. In the ROSE group, the diagnosis rate of adenocarcinoma is reduced compared with pathological results (34.1% vs. 25.8%), and the difference is statistically significant. However, for SCLC, no statistically significant difference is noted for the diagnosis rates between the ROSE group and the pathological diagnosis. For SCC, ROSE was moderately consistent with the pathological results, and no statistically significant difference in the diagnosis rates was noted between the ROSE group and the pathological results. In the ROSE group, 2 cases received a diagnosis of cancer suspicion, and the final pathological diagnosis was atypical hyperplasia. In addition, in our study, the main infectious diseases included pulmonary inflammation, tuberculosis, and fungal infections. No significant difference in diagnostic rates was noted between the ROSE group and pathological results, but ROSE exhibited an increased diagnostic rate for pulmonary inflammation, tuberculosis, and fungal infections. Therefore, we believe that CT-guided percutaneous FNA combined with ROSE exhibits an accurate diagnosis rate and that the ROSE and the pathological diagnosis results are consistent. ROSE can be used to estimate the type of disease at an early stage and further assist in the identification and adjustment of early treatment.

The complications of CT-guided percutaneous FNA mainly include pneumothorax and hemoptysis, which are mostly mild with a low incidence. Other serious complications, such as air embolism, cancer cell spread via needle, cardiac and respiratory arrest, and shock, are rare [26–29]. The results of studies on the complications of CT-guided percutaneous FNA combined with ROSE are also inconsistent. Some studies report complications of less than 2% [16, 30]; however, some studies report complications in 20% or up to 50% of cases [31–35]. In our study, the incidence of complications in the ROSE group was 8.3%. Specifically, the incidences of pneumothorax and hemoptysis are 6.8% and 1.5%, respectively. Thus, the incidence of complications in our study is low. The incidence of complications in the NO-ROSE group is 16.7%. Specifically, the incidence of pneumothorax and hemoptysis was 14.7% and 2.0%, respectively. No statistically significant differences in the incidence of complications were noted with or without ROSE (*P* > 0.05). In our study, no serious complications, such as air embolism, pericardial tamponade, or cancer cell metastasis via the needle pathway, occurred. No severe consequences occurred in all patients after supportive treatment for complications was provided, which is consistent with current studies [9, 12, 36, 37].

Our research also has some limitations: (1) we exclusively conducted our study at Taihe Hospital, and other institutions were not included. Thus, the results may be biased. (2) This is a retrospective study. Thus, the data may be biased, and some data could not be reviewed as the record was not available. (3) We cannot control the impact of the proficiency of all operators on the results.

All in all, we believe that ROSE, which requires simple equipment and processes, is a safe, quick, and effective means of ancillary examination. ROSE offers high diagnostic accuracy, sensitivity, and specificity when applied to CT-guided percutaneous FNA for pulmonary occupying lesions, especially adenocarcinoma and small cell lung cancer given that ROSE is considered consistent with the pathological diagnosis. CT-guided FNA diagnosis with ROSE did not increase the risk of complications, such as pneumothorax and hemoptysis, so this technique should be used in the clinic.

## Figures and Tables

**Table 1:** Characteristics of the two groups of patients.

Variables	With ROSE (*n* = 132)	Without ROSE (*n* = 102)	*P* value
Age (years)	57.34 ± 11.658	56.40 ± 11.476	0.539
Gender (male/female)	86/46	66/36	0.994

**Table 2:** Final pathological diagnosis results of CT-guided percutaneous FNA with or without ROSE.

Result		ROSE group (ROSE results)	NO-ROSE group
Not cancer	Inflammation	30 (31)	22
	Tuberculosis	24 (34)	19
	Fungal infection	3 (1)	1
	Atypical hyperplasia	5 (2)	1
	Other	6 (4)	5

Malignancy	AC	45 (34)	34
	SCC	11 (6)	9
	NSCLC-NOS	1 (12)	5
	SCLC	3 (3)	1
	Other	4 (5)	3

Benign	Hamartoma	0 (0)	2

Total		132	102

AC: adenocarcinoma; SCC: squamous cell carcinoma; NSCLC-NOS: nonsmall cell lung cancer-not; SCLC: small cell lung cancer.

**Table 3:** Comparison of the ROSE result with the final pathological diagnosis of patients using FNA (*n* = 132).

ROSE result	Final pathological diagnosis		Total
	Cancer	Not cancer	
Cancer	57	4	61
Not cancer	7	64	71
Total	64	68	132

**Table 4:** Cytological malignant diagnoses in the ROSE group compared with the final pathological result.

Malignancy	ROSE group	Final pathological result
AC	34	45
SC	6	11
NSCLC-NOS	12	1
SCLC	3	3
Other malignancy	5	4
Total	61	63

**Table 5:** Correlation between the ROSE result and pathology in malignant cases (*n* = 132).

ROSE result	Final pathological diagnosis						Total
	AC	SCC	NSCLC-NOS	SCLC	Other malignancy	Not cancer	
AC	33	0	0	0	0	1	34
SCC	0	4	0	0	2	0	6
NSCLC-NOS	7	4	1	0	0	0	12
SCLC	0	0	0	3	0	0	3
Other malignancy	1	0	0	0	2	2	5
Not cancer	4	3	0	0	0	65	72
Total	45	11	1	3	4	68	132

**Table 6:** Pulmonary infection diagnoses in the ROSE group compared with the final pathological result.

Category	ROSE group	Final pathological result
Inflammation	31	30
Tuberculosis	34	24
Fungal infection	1	3
Total	66	57

**Table 7:** Correlation between the ROSE result and pathology in pulmonary infection cases (*n* = 132).

ROSE result	Final pathological diagnosis				Total
	Inflammation	Tuberculosis	Fungal infection	No infection	
Inflammation	24	0	1	6	31
Tuberculosis	6	23	1	4	34
Fungal infection	0	0	1	0	1
No infection	0	1	0	65	66
Total	30	24	3	75	132

## Data Availability

All data generated or analyzed during this study are included in this article. The datasets used and/or analyzed during the current study are available from the corresponding author on reasonable request.
